# Syringomatous tumor of the nipple: a case report

**DOI:** 10.11604/pamj.2024.48.1.37845

**Published:** 2024-05-02

**Authors:** Ana Vitória de Jesus Félix, Larissa Vasconcelos Silva, Rafael Everton Assunção Ribeiro da Costa, Maria Júlia Andrade Pereira Soares, Raniere Francisco de Oliveira Sobrinho, Maria Clara Amorim Silva, Raimundo Gerônimo da Silva Júnior, Sabas Carlos Vieira

**Affiliations:** 1 Health Science Center, Federal University of Piauí, Teresina (PI), Brazil,; 2 Health Science Center, State University of Piauí, Teresina (PI), Brazil,; 3 Medical School, UNINOVAFAPI University Centre, Teresina (PI), Brazil,; 4 Obstetrics and Gynecology, Oncocenter, Teresina (PI), Brazil

**Keywords:** Breast tumors, nipples, pathology, case report

## Abstract

Syringomatous tumor of the nipple is a benign, locally infiltrative tumor. There are reports in the literature of tumor recurrence in cases of incomplete excision. Clinical and mammographic findings in syringomatous tumors are like those of breast carcinoma and the pathologist has a fundamental role in final tumor diagnosis. Therefore, the aim of this study was to report a case of syringoma located in the areolar region. A 33-year-old woman reported that she had noticed a nodule in her left areolar region 4 years previously (February 2019). A breast ultrasound was performed, detecting intraparenchymatous breast cysts. Surgical resection of the nodule was indicated although it was not performed. Two years later, in August 2021, the patient underwent a mastopexy with prosthesis inclusion. Histopathology study of the surgical specimen revealed a syringomatous tumor with positive margins. Thirteen (13) months after diagnosis (September 3, 2021 - October 16, 2022), the patient is doing well and receives clinical follow-up.

## Introduction

Syringomatous adenoma of the nipple was first reported in 1983 by Rosen. It is a benign breast tumor that is histologically like syringoma and shows local infiltrative proliferation. This type of tumor is rare, and it may be difficult to diagnose. Furthermore, although it is a locally infiltrative tumor with no evidence of distant metastases, recurrences have been reported in cases of incomplete excision. In 2019, the World Health Organization (WHO) recommended syringomatous tumor of the nipple for nomenclature of this tumor [1]. Syringomatous tumor of the nipple is usually misdiagnosed, since its clinical and mammographic findings mimic carcinoma. It may be confounded with tubular carcinoma and low-grade adenosquamous carcinoma of the breast. It is thus fundamental that physicians and pathologists recognize this type of tumor to avoid misdiagnosis and unnecessary treatment [2]. Furthermore, it is fundamental to provide adequate treatment to avoid relapses. In one of the largest series published, 45% of the patients had a recurrence after local excision [3]. The aim of this study is to report a case of syringomatous tumor of the nipple.

## Patient and observation

**Figure 1:**
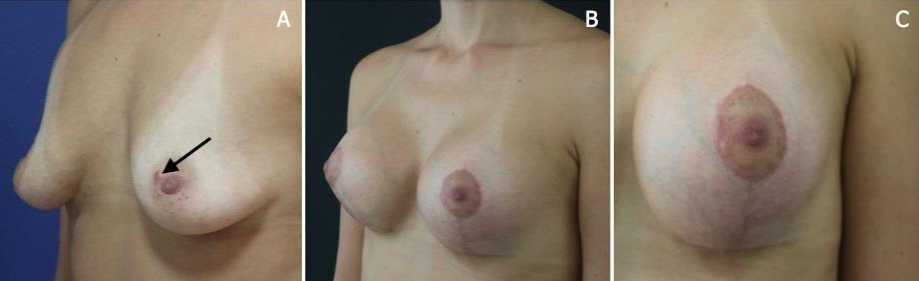
A) lesion in the left nipple (indicated by the arrow); B) aspect of the breast after mastopexy with prosthesis inclusion; C) final aspect of the nipple after excision of the lesion

**Patient information:** a 33-year-old female patient reported that she had noticed a small nodule in the areola of her left breast 4 years previously (February 2019), (Figure 1A), located at the 11 o'clock position, without any other prior disease or previous pregnancies. Her brother had been diagnosed with leukemia. The patient had a history of surgical resection of a right arm lesion, described as ossifying myositis that progressed without sequelae.

**Clinical findings**: the patient had a mobile nodule that was smaller than 1 cm in the medial region of her left nipple. It was restricted to the skin and was clinically consistent with epidermal cyst.

**Figure 2:**
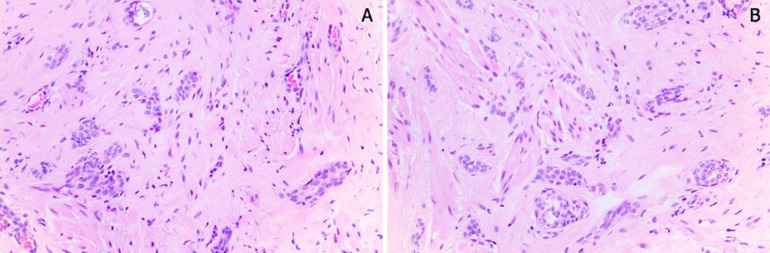
A,B) hematoxylin-eosin (HE) histopathology study (magnification of x 100 in both slides)

**Diagnostic assessment:** on ultrasound, the patient only had intraparenchymatous breast cysts, with no mention of skin lesions. Histopathological study of the surgical specimen showing tumor composed of cords of uniform cells and without atypia, some with irregular shapes and infiltrative aspect revealed an atypical epithelial lesion (Figure 2). Immunohistochemistry showed ER negative, PR negative, HER-2 negative, ki-67 positive tumor in rare cells, p63 negative and SMMS negative.

**Diagnosis:** pathology results show a syringomatous tumor with positive margins.

**Therapeutic interventions:** resection of the nodule was indicated, although it was not done. After 2 years (August 2021), the patient underwent a mastopexy with prosthesis inclusion (Figure 1B). During surgery, the areolar nodule was resected.

**Follow-up and outcome of interventions:** the case was discussed in multidisciplinary meeting and patient follow-up was chosen. The patient is currently (Figure 1C) doing well and is under periodical consultations in the oncology center.

**Informed consent:** the study was approved by the Research Ethics Committee of the Federal University of Piauí (CEP-UFPI), with report 5.382.151. The patient signed the free informed consent term (FICT).

## Discussion

Syringomatous tumor of the nipple (SyT) is a rare type of tumor that usually manifests itself as a solitary unilateral lesion of the nipple areolar complex, measuring 1 to 3 cm in diameter. Fewer than 60 cases of the disease have been reported. It is an underdiagnosed tumor, due to its rare appearance and similarity to other types of tumors. SyT occurs in patients ranging from 11 to 76 years of age. Mean age at the time of presentation is 40 years of age. Nipple discharge may occur, but it is uncommon. However, the presence of tenderness and itchiness are more common symptoms. Macroscopically, these masses are usually irregular and less well-defined, brownish, or gray. Nevertheless, these tumors may also manifest as well-defined masses. It is common for these tumors to appear as a firm solitary mass in the subareolar or mammillary region as occurred in the current case described. Nevertheless, these tumors may also occur within the breast parenchyma [4]. Findings in imaging diagnostic tests for SyT may be quite like those of malignant tumors, therefore is difficult to differentiate SyT from carcinomas by tests only, such as ultrasonography, mammography, and magnetic resonance imaging. Ultrasound of SyT shows a solid poorly defined nodule with heterogenous internal echoes. On mammography, SyT usually appears as a high-density lesion in the subareolar region with irregular shape, formation of spindles and foci of microcalcifications [2].

Due to these similarities, a histological exam is required for diagnosis of SyT. Histologically, this type of tumor has elongated, irregular infiltrative ducts and tubules, involving smooth muscle bundles of the surrounding deep dermis and nipple stroma, with the shape of a comma or teardrop, which helps differentiate this tumor from other lesions [5]. The main differential diagnoses include adenoma of the mammillary duct, well-differentiated tubular carcinoma, and Paget´s disease of the nipple. In general, adenoma of the nipple causes skin ulceration. It is more circumscribed and does not involve smooth muscle bundles. In contrast, tubular carcinoma tends to occur more deeply in the outer upper quadrant of the breast. If the tubular carcinoma extends to the nipple, it may cause nipple retraction and Paget´s disease [6]. Optimal early management of SyT is complete tumor resection with histologically negative margins since there may be local tumor recurrence in incompletely excised lesions. Therefore, a new excision is recommended in case of positive tumor margins. Nevertheless, metastatic disease in SyT patients has not been described in the literature to date [6].

## Conclusion

The current case shows that despite positive tumor margins, the patient refused to be reoperated after a broad discussion with the surgical team. Thirteen (13) months after the diagnosis was made (September 3, 2021 - October 16, 2022), she still has no evidence of local recurrence and continues to receive periodical clinical follow-up at the oncology center.
